# Comparison of two different TSH-receptor antibody assays: A clinical practice study

**DOI:** 10.1016/j.heliyon.2023.e22468

**Published:** 2023-11-18

**Authors:** Mathé van Balkum, Marco W.J. Schreurs, W. Edward Visser, Robin P. Peeters, Willem A. Dik

**Affiliations:** a Department of Internal Medicine, the Netherlands; b Laboratory Medical Immunology, Department of Immunology, Erasmus MC, University Medical Center, Rotterdam, the Netherlands; c Academic Center for Thyroid Diseases, Dr. Molewaterplein 40, 3015 CE, Rotterdam, the Netherlands

**Keywords:** Graves' disease, Graves orbitopathy, TSH-Receptor auto-antibody, TRAb, Thyrotropin binding inhibiting immunoglobulins, TBII, Thyrotropin stimulating immunoglobulins, TSI

## Abstract

**Background:**

Graves’ disease (GD) is caused by the production of TSH-receptor (TSHR) stimulating auto-antibodies. Over the years various TSHR-antibody (TRAb) detection assays have been developed. Most clinical laboratories use competitive TSH-binding inhibitory immunoglobulin (TBII) assays, which measure the total amount of stimulating and blocking auto-antibodies. Selective detection of TSHR stimulating auto-antibodies (TSI) was previously only possible with functional cell-based bioassays. However, more recently an automated bridge-based binding assay to more specifically measure TSI has become available. The aim of our study was to compare the third-generation automated competitive immunoassay (TBII) with the automated bridge immunoassay (TSI) in clinical practice in an academic thyroid expert center.

**Methods:**

A retrospective study in 356 patients with Graves’ disease, Graves orbitopathy (GO), and other (thyroid) disease treated in an academic thyroid center was performed. All samples were analyzed for TBII and TSI. For both assays, sensitivity, specificity, positive predictive value (PVV), negative predictive value (NPV) and diagnostic odds ratios were calculated using different cut-offs for negativity.

**Results:**

Using the provided cut-off, the overall sensitivity appeared similar between TBII and TSI, but TSI showed higher overall specificity, PPV, NPV and diagnostic odds ratio. Using two or three times the cut-off for negativity resulted in a decrease in sensitivity, but an increase in specificity and PPV, which was most pronounced for the TBII-assay. Analysis in a subgroup of newly diagnosed treatment naïve GD/GO patients also revealed overall favorable results for the TSI-assay. Increasing the cut-off for negativity resulted in increased specificity for both assays, with similar results using two or three times the cut-off. Most patients with concordant positive results for TBII and TSI suffered from GD or GD + GO (n = 110, 95.6 %), while patients negative for both TBII and TSI mostly suffered from other (thyroid) disease (n = 143, 77.3 %). From patients with positive TBII but negative TSI only 42.1 % had GD/GO (n = 16), whereas 57.9 % (n = 22) had other (thyroid) disease. In contrast, 88.9 % of patients with positive TSI but negative TBII had GD/GO (n = 16), whereas 11.1 % (n = 2) had other (thyroid) disease.

**Conclusion:**

In our academic thyroid center, the diagnostic performance of the TSI-assay outperformed the TBII-assay. Using a higher cut-off value for negativity can be helpful in assessing clinical relevance.

## 1 Introduction

Hyperthyroidism is a common condition with a prevalence of approximately 1.2 % worldwide and is characterized by inappropriately high concentrations of circulating thyroid hormones [1,2]. Graves’ disease (GD) is the most common underlying cause (50–80 % of cases) [2,3]. GD is an autoimmune disorder with an incidence of 20–30 cases per 100.000 individuals per year and is more prevalent in women [4]. Hyperthyroidism in GD is caused by circulating auto-antibodies that stimulate the TSH-receptor (TSHR), leading to unregulated production and secretion of thyroid hormones [2,5,6]. Measurement of these TSH-receptor antibodies (TRAb) in patient serum is a sensitive tool to diagnose GD. Functionally TRAb can be divided into two categories: 1) thyroid stimulating antibodies (TSAb; TSI) and 2) thyroid blocking antibodies (TBAb; TBI), that can both (co)-exist in patients with GD [[7], [8], [9]].

Besides diagnosis, there are several other clinical implications where TRAb measurement is of added value. TRAb normally decline during treatment with antithyroid drugs (ATD) and can therefore be used to monitor disease course. However, in a substantial percentage of patients with GD, remission is not achieved or patients experience relapse after stopping ATD [[1], [2], [3]]. Importantly, high TRAb concentrations, prior to treatment, are associated with a higher relapse rate following ATD [1,2,[10], [11], [12]]. Therefore, TRAb testing can be of value to predict sustained disease remission or relapse before ATD is stopped [13]. Furthermore, high TRAb levels are associated with increased risk for developing Graves’ orbitopathy (GO), a complication where the soft orbital tissues are the target of autoimmune attack by TRAb and other immune components [3,6,[14], [15], [16]]. In addition, serum TRAb concentration, especially of TSI, strongly correlates with the clinical activity score (CAS) of GO and can therefore be used in the follow-up and to optimize timing of rehabilitative surgery [[17], [18], [19], [20], [21]]. Finally, in pregnant women with GD TRAb monitoring is important as these antibodies are transported across the placenta during the last pregnancy trimester and thereby can cause severe complications like fetal hyperthyroidism and neonatal graves disease [[22], [23], [24]].

Various types and generations of TRAb detection assays have been developed over the years. Most routine clinical laboratories currently use competitive binding assays to detect TRAb. These assays measure the inhibition of binding of a ligand to TSH-R, referred to as TSH-binding inhibitory Immunoglobulin (TBII) assays. Although currently used generations of this type of assay display high clinical sensitivity (>97 %) and specificity (98–99 %) rates, they detect both TSI and TBI and provide no information on functional properties of the detected TRAb [25]. Selective detection of stimulating antibodies (TSI) is possible with technically more challenging functional cell-based bioassays [26]. More, recently a novel automated bridge-based binding assay has become commercially available to detect TSI more selectively, although not exclusively since some TBI can bind to the capture TSH-receptor chimera used in this bridge-based assay [[27], [28], [29], [30]]. Previous studies reported high sensitivity and specificity rates varying from 81 to 100 % and 73–100 %, respectively, for this automated bridge-based binding assay in diagnosing GD [27,28,[31], [32], [33], [34], [35], [36], [37]]. Where most studies investigated the overall correlation between the different assay methods, only few studies included clinical characteristics in relation to this outcome [27,31,32,34,36]. Most hospitals, including our academic thyroid center, use a competitive binding assay to detect TRAb. In the current study, we compared the clinical performance and relevance of the TBII-assay and the more novel bridge-based TSI-assay in patients seen in our academic thyroid center, especially in relation to clinical disease characteristics.

## 2 Materials and methods

### 2.1 Study design and patients

This retrospective clinical practice study was approved by the medical ethical committee of Erasmus MC (MEC-2016-606) and performed in all patients who had routine TRAb testing in our academic thyroid center (Erasmus MC, Rotterdam) from April 2019 to July 2020. All patients in whom TRAb measurement was conducted during this period were included, regardless of their underlying diagnosis or time point in treatment course. From patients who had several TRAb measurements during the inclusion period, only the first sample was included in this study. The diagnosis of GD was based on clinical characteristics, presence of (sub)clinical hyperthyroidism, documented TRAb positivity or findings on I-123 scintigraphy. If GO was present, we included the most recent ( ± 3 months) CAS according to the standardized criteria of the European Group on Graves Orbitopathy (EUGOGO) [38]. The CAS could either be determined at our thyroid outpatient clinic or by the ophthalmologist. GO was considered active when CAS ≥3. Patients <18 years were excluded from the study.

### 2.2 TRAb measurement

In all serum samples in which a routine clinical TRAb measurement was requested this was performed with an automated competitive TBII fluorescent enzyme-immunoassay (EliA anti-TSH-R, Thermofisher Scientific, Freiburg, Germany). Hereafter, samples were stored at −20 °C for a maximum of two weeks before being re-analyzed with the bridge-based TSI binding assay (Immulite 2000 TSI; Siemens Healthcare diagnostics, Llanberis, UK). For the TBII-assay the manufacturer defined <2.9 IU/L as negative and designed the range 2.9–3.3 IU/L as equivocal, which was also confirmed by an internal validation of the Laboratory Medical Immunology (LMI) at Erasmus MC. Therefore, for the current study we used <2.9 IU/L as cut-off for negativity. TBII values above 68 IU/L were reported as >68. The cut-off for negativity for the bridge-based TSI binding assay as defined by the manufacturer is < 0.55 IU/L, with a detection range of 0.1–40.0 IU/L. TSI values above 40 IU/L were reported as >40. The LMI was blinded for patients history and diagnosis during analysis of the samples with both assays.

### 2.3 Statistical analyses

Statistical analysis was performed with IBM SPSS Statistics 25. Categorical data were presented as numbers with percentage, and compared with Fisher's or Pearson's chi-squared test. For continuous, not-normally distributed data the medians and interquartile range (IQR) were calculated and outcomes were compared with the Mann-Whitney *U* test. As TBII and TSI outcomes were not normally distributed, Spearman's correlation coefficient was calculated to assess the correlation between the two assays. Clinical sensitivity, specificity, positive predictive value (PVV) and negative predictive value (NPV) were calculated for both assays to determine the diagnostic accuracy of the tests. Furthermore, diagnostic odds ratios (DOR) were calculated as a single indicator of test performance in relation to the test's accuracy. Higher DOR values indicate better discriminatory test performance [39]. Analyses were performed for both the total study population as well as for a subgroup of treatment naive patients. In addition, to exclude most false positive results and improve clinical relevance, we also carried out analyses using two and three times the cut-off values for negativity. These cut-offs are based on national and international guidelines for pregnant women that recommend close monitoring when TRAb level exceeds the upper limit of normal (cut-off) by more than 2–3 fold [23,24,40,41]. Differences with p value < 0.05 were considered statistically significant.

## 3 Results

### 3.1 Descriptive data

**Fig. 1:**
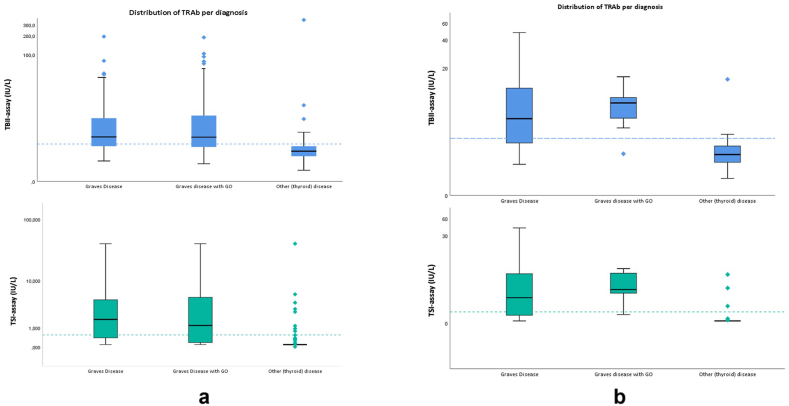
ADistribution of TRAb outcomes using the two different assays in the total study population on a logarithmic scale. The dashed-line represents the cut-offs for negativity (<2.9 IU/L for the ELiA TBII assay and <0.55 IU/L for the Immulite TSI assay). Fig. 1b. Distribution of TRAb outcomes using the two different assays in the treatment naïve cohort with abnormal thyroid function tests on a logarithmic scale. The dashed-line represents the cut-offs for negativity (<2.9 IU/L for the ELiA TBII assay and <0.55 IU/L for the Immulite TSI assay).

**Fig. 2:**
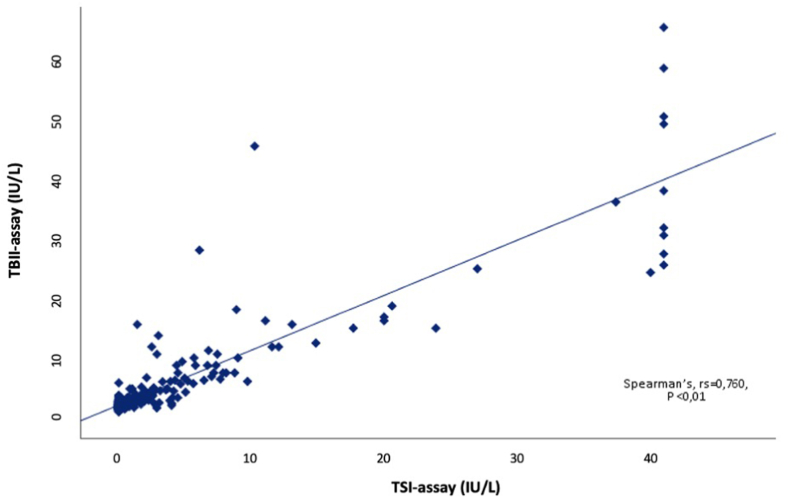
Correlation analysis in the total study population between the TBII-assay and the TSI bridge-based binding assay. A positive correlation was seen between the two assays for all samples analyzed (Spearman's, rs = 0.760, P < 0.01).

A total of 356 patients were included for analysis in this study. Table 1 shows the characteristics of the 184 included Graves' disease patients (N = 90 GD and N = 94 with GD + GO) and 172 individuals with other (thyroid) disease. For the group with other (thyroid) disease further information on underlying diseases is provided in Supplemental 1a. Among the 184 GD/GO patients, 31 patients were newly diagnosed treatment naïve (abnormal thyroid function tests i.e. hyperthyroidism or subclinical hyperthyroidism). From the GD + GO patients, 19 had active, moderate-to-severe, disease (CAS ≥3). As expected, GD patients showed significantly higher TRAb values than patients with other (thyroid) disease. The distribution of TRAb levels measured with the TBII-assay and the TSI-assay for both the total study population and the treatment naïve cohort are depicted in Fig. 1a and b, respectively. Within the subgroup of treatment naïve newly diagnosed GD/GO patients the median TBII-level was 5.9 ± 8.3 IU/L and the median TSI-level was 2.19 ± 6.0 IU/L. The median TBII level in the active-GO-group was 5.8 ± 21.8 IU/L, whereas the median for TSI was 2.34 ± 5.9 IU/L. Overall, a positive correlation was seen between the TBII-assay and TSI-assay (Spearman's, rs = 0.760, P < 0.01; Fig. 2).

**Table 1:** Characteristics of the study population.

	GD/GO (N = 184)^a^	Other (thyroid) disease (N = 172)^a^	P-value^b^
Age years, median	50 (18–80)	47 (18–93)	0.446
Sex, female (%)	143 (77.7 %)	132 (76.9 %)	0.634
Thyroid function (%)			0.113
- Euthyroidism	75 (40.8 %)	86 (38.2 %)	
- Hyperthyroidism	58 (31.5 %)	58 (25.8 %)	
- Subclinical hyperthyroidism	36 (19.6 %)	38 (16.9 %)	
- Subclinical hypothyroidism	10 (5.4 %)	25 (11.1 %)	
- Hypothyroidism	3 (1.6 %)	12 (5.3 %)	
- Low TSH and low free T4	2 (1.1 %)	–	
- Unknown	–	6 (2.7 %)	
TSH, median (mIU/l)	0.42 ± 2.0 (<0.003–53.8)	0.61 ± 2.4 (<0.003–127.9)	**0.029**
- M. Graves	0.07 ± 1.4 (<0.003–53.8)	–	
- M. Graves with GO	0.70 ± 2.1 (<0.003–6.1)	–	
Free T4, median (mIU/l)	22.9 ± 8.8 (6.8–173.8)	20.9 ± 10 (2.3–136.6)	**0.015**
- M. Graves	23.5 ± 14.7 (6.8–173.8)	–	
- M. Graves with GO	22.4 ± 7.3 (14.2–122)	–	
Therapy (%)			**<0.001**
- No current treatment	55 (29.9 %)	119 (69.2 %)	
- B&R therapy	55 (29.9 %)	–	
- Monotherapy	21 (11.4 %)	–	
- LT4 suppletion	50 (27.2 %)	39 (22.7 %)	
- Immunosuppression	3 (1.63 %)	2 (1.2 %)	
- Unknown	–	12 (6.9 %)	
RAI in past history (%)	46 (2.1 %)	8 (4.7 %)	**<0.001**
TBII EliA, median (range) (IU/L)	4.00 ± 7.1 (0.9–195)	2.0 ± 1.2 (0.5–358)	**<0.001**
- M. Graves	4.10 ± 6.5 (1.1–195)		
- M. Graves with GO	4.00 ± 7.8 (0.9–189)		
TSI Immulite, median (range) (IU/L)	1.32 ± 4.5 (0.09->40)	0.1 ± 0.0 (<0.1 - >40)	**<0.001**
- M. Graves	1.81 ± 4.3 (<0.1 - >40)		
- M. Graves with GO	1.18 ± 5.0 (<0.1- >40)		

a Data is presented as numbers (percentage of the respective population) or medians (IQR).

b p-value comparing patients with GD/GO and other (thyroid) disease. P-values <0.05 were considered significant.

### 3.2 Diagnostic accuracy

The diagnostic accuracy of both tests for diagnosing GD (based on the cut-off values as provided by the manufacturers) is given in Table 2a. Although the sensitivity appeared similar between TBII and TSI (67.9 %), TSI showed higher specificity, PPV, NPV and DOR. At the cut-off value provided by the manufacturer the estimated DOR for TSI was higher (43 vs 12), indicating a better performance for TSI in diagnosing GD.

**Table:** 2aDiagnostic accuracy in total study population with GD/GO vs other (thyroid) disease (n = 356) using different cut-off for test negativity.

Test	Sensitivity %	Specificity %	PPV %	NPV %	DOR
TRAb TBII ELiA					
- Cut-off <2.9 IU/L	67.9 %	84.9 %	82.8 %	71.2 %	12
- Cut-off <5.8 IU/L	36.4 %	98.3 %	95.7 %	59.1 %	32
- Cut-off <8.7 IU/L	27.7 %	98.8 %	96.2 %	56.1 %	33
TRAb TSI Immulite					
- Cut-off <0.55 IU/L	67.9 %	95.3 %	94.0 %	73.5 %	43
- Cut-off <1.1 IU/L	54.9 %	96.5 %	94.4 %	66.7 %	34
- Cut-off <1.65 IU/L	47.3 %	97.1 %	94.6 %	63.3 %	30

PPV = positive predictive value, NPV = negative predictive value, DOR = diagnostic odds ratio.

Using two or three times the cut-off for test negativity resulted in a decrease in sensitivity, but an increase in specificity and PPV, which was most pronounced for the TBII assay. A subgroup analysis was performed on newly diagnosed treatment naïve GD/GO patients. This also revealed overall favorable results for the TSI-assay in diagnosing GD when the manufacturers cut-off values were applied (Table 2b). Increasing the cut-off for test negativity resulted in increased specificity for both assays, with similar results using two or three times the cut-off.

**Table 2b:** Diagnostic accuracy in treatment naïve GD/GO group vs other (thyroid) disease^a^ using different cut-off for test negativity.

Test	Sensitivity %	Specificity %	PPV %	NPV %	DOR
TRAb TBII ELiA					
- Cut-off <2.9 IU/L	71.0 %	90.4 %	81.5 %	83.9 %	23
- Cut-off <5.8 IU/L	51.6 %	98.1 %	94.1 %	77.3 %	54
- Cut-off <8.7 IU/L	32.3 %	98.1 %	90.9 %	70.8 %	24
TRAb TSI Immulite					
- Cut-off <0.55 IU/L	74.2 %	94.2 %	88.5 %	86.0 %	47
- Cut-off <1.1 IU/L	67.7 %	96.2 %	91.3 %	83.3 %	53
- Cut-off <1.65 IU/L	58.1 %	96.2 %	90.0 %	79.4 %	35

PPV = positive predictive value, NPV = negative predictive value, DOR = diagnostic odds ratio.

a Only untreated patients with abnormal thyroid function tests (i.e subclinical hyperthyroidism or hyperthyroidism) were included.

### 3.3 Comparison of concordance between both TRAb assays

In the total study population (N = 356), most patients with concordant positive results for TBII and TSI suffered from GD or GD + GO (n = 110, 95.6 %), while patients negative for both TBII and TSI mostly suffered from another (thyroid) disease (n = 143, 77.3 %) (Table 3a). From the patients with positive TBII but negative TSI only 42.1 % had GD/GO (n = 16), whereas 57.9 % (n = 22) had other (thyroid) disease. In contrast, in patients with positive TSI but negative TBII 88.9 % had GD/GO (n = 16), whereas 11.1 % (n = 2) had other (thyroid) disease. Both in the treatment naïve group (N = 31) and group of patients with active GO (N = 19) most patients showed TRAb positivity in both assays (67.7 % and 68.4 %, respectively. Tables 3b and 3c). There were 42 patients with TBII-levels in the grey zone area of 2.9–3.3 IU/L. Most of them had negative TSI-levels (N = 27; 64.3 %) and suffered from other (thyroid) pathologies (17/27, 63 %). From the 15 patients with equivocal positive results with TBII but positive results with TSI, 13 patients had GD/GO (86.7 %) and 2 (13.3 %) had other (thyroid) disease.

**Table:** 3aSerological outcomes in total study population.^a^.

	M. Graves (N = 90)	M. Graves with GO (N = 94)	Other diagnosis (N = 172)
TBII and TSI both negative	21 (11.4 %)	21 (11.4 %)	143 (77.3 %)
TBII and TSI both positive	55 (47.8 %)	55 (47.8 %)	5 (4.3 %)
TBII positive and TSI negative	5 (13.2 %)	11 (28.9 %)	22 (57.9 %)
TBII negative and TSI positive	9 (50.0 %)	7 (38.9 %)	2 (11.1 %)
**Outcome**	Concordant 76/90 = 84.4 % Discordant 14/90 = 15.6 %	Concordant 76/94 = 80.9 % Discordant 18/94 = 19.1 %	Concordant 148/172 = 86.0 % Discordant 24/172 = 14.0 %

a Cut-off for negativity for TBII-Assay (Elia) < 2.9 IU/L and TSI-assay (Immulite) < 0.55 IU/L.

**Table 3b:** Serological outcomes in treatment naïve group a^,^^b^.

	M. Graves (N = 24)	M. Graves with GO (N = 7)	Other diagnosis (N = 52)
TBII and TSI both negative	6 (25 %)	1 (14.3 %)	45 (86.5 %)
TBII and TSI both positive	15 (62.5 %)	6 (85.7 %)	1 (1.9 %)
TBII positive and TSI negative	1 (4.2 %)	–	5 (9.6 %)
TBII negative and TSI positive	2 (8.3 %)	–	1 (1.9 %)
**Outcome**	Concordant 21/24 = 87.5 % Discordant 3/24 = 12.5 %	Concordant 7/7 = 100 % Discordant -	Concordant 46/52 = 86.5 % Discordant 6/52 = 11.5 %

a Treatment naïve group = Untreated patients with abnormal thyroid function tests (i.e. subclinical hyperthyroidism or hyperthyroidism).

b Cut-off for negativity for TBII-Assay (Elia) < 2.9 IU/L and TSI-assay (Immulite) < 0.55 IU/L.

**Table 3c:** Serological outcomes in active GO group^a^^,^^b^.

	M. Graves with active GO (N = 19)
TBII and TSI both negative	4 (21.1 %)
TBII and TSI both positive	13 (68.4 %)
TBII positive and TSI negative	–
TBII negative and TSI positive	2 (10.5 %)
**Outcome**	Concordant 17/19 = 89.5 % Discordant 2/19 = 10.5 %

a Active GO group = CAS ≥3 according to EUGOGO.

b Cut-off for negativity for TBII-Assay (Elia) < 2.9 IU/L and TSI-assay (Immulite) < 0.55 IU/L.

Supplemental Tables 2a and 2b shows results obtained when two and three times the cut-off values for negativity were used. Increasing the cut-off by two or three-fold resulted in increased numbers of antibody negatives in the GD/GO group in both assays. Interestingly, when using two times the cut-off, only 3 patients (1.6 %) in de GD/GO group were TBII positive and TSI negative, whereas 37 (20.1 %) were only positive with the TSI-assay and negative with the TBII-assay. Similar results were seen using three times the cut-off, with only 1 patient (0.5 %) in the GD/GO group displaying only TBII positivity and 37 patients (20.1 %) with TBII negative and TSI positive results. In the subgroup with other (thyroid) disease, using two or three times the cut-off, resulted in a higher concordance rate of 98.3 %. Patients with positive TRAb tests but other (thyroid) disease than GD are shown in Supplemental Table 3.

## 4 Discussion

Over the last years various types and generations of assays have been developed for TRAb detection, and clinicians have to be aware of the type of assay used for TRAb measurement and their limitations [25]. The objective of this study was to compare two different TRAb-binding assays in a daily practice study in a large cohort of patients with GD/GO and other (thyroid) disease that visit an academic thyroid center. Our data clearly shows that in this academic setting the bridge-based TSI-assay showed better diagnostic accuracy for both the total study population and treatment naïve group than the more commonly used TBII-assay. Increasing the cut-off value for assay negativity by two or three times improved clinical relevance with an increased specificity and PPV, with similar results using two or three times the cut-off. For the TSI-assay, m ost previous studies reported an ideal cut-off around the cut off proposed by the manufacturer to maximize sensitivity and specificity [28,31,32]. The sensitivity rates in our study are lower than described in previous studies, with sensitivities varying from 81 to 100 % [27,[31], [32], [33], [34], [35], [36], [37]]. This can be explained by the daily practice approach we applied for our study. We included all patients with a TRAb measurement during a routine clinical visit and thus independent from their timepoint in disease course. It is well known that TRAb levels decline gradually under ATD treatment and disappear in a large proportion of patients after 12–18 months of treatment [53,54]. Our results are more in line with the study from Silvestre et al. [36], where they determined an ideal cut-off of 1.42 IU/L for the TSI-test with a sensitivity of 73 % and specificity of 89 %. A possible explanation is that, comparable to our study, they also included patients with different types of thyroid pathologies instead of healthy volunteers. Subgroup analysis of only newly diagnosed treatment naïve patients in our study did increase the sensitivity of both assays, although this was still lower than reported in previous studies. The high specificity rates for the TSI-assay in our study are in line with the specificity rates from previous studies, where in most studies a specificity >90 % is reported [27,28,[32], [33], [34],^37^]. Only Silvestre et al. [36] and Scappaticcio [35] reported lower specificity rates of 73 % and 84.2 %, respectively. For the TBII-assay we denoted patients with equivocal positive results (the grey zone for positivity: 2.9–3.3 IU/L) as TBII-positive, which influences sensitivity and specificity calculations of this test in our study. The sensitivity and specificity (71 % and 90.4 %, respectively) in the newly diagnosed treatment naïve group are in line with the results from Struja et al. [37] who reported a sensitivity of 79.5 % and specificity of 93.7 % using the original cut-off of 2.9 IU/L, and a sensitivity of 71.1 % and specificity of 97.9 % when a cut-off of 3.3 IU/L was applied. The results are also similar with the results from the manufacturer where they found a sensitivity varying between 83 % at a cut-off of 2.9 IU/l and 79 % at a cut-off of 3.3 IU/l [46]. These two forementioned studies also included patients with different types of thyroid pathologies rather than healthy volunteers. Including different thyroid pathologies may better reflect real life indications for TRAb testing and is therefore considered a strength of our study.

In the total study population most patients (95.6 %) had concordant results for TBII and TSI, while this was the case for 90.3 % of patients in the newly diagnosed treatment naïve GD group. The discordance rate of 9.7 % is higher than reported in the study from Scappaticcio et al. where they reported a discordance rate of 1.2 % in patients with GD and 2.6 % in patients with non-graves hyperthyroidism [35]. In total, 7 sera of patients in the group of newly diagnosed treatment naïve GD remained negative in both assays. Lack of detectable TRAb at initial presentation, or during follow-up, has previously been noted in some GD patients [[42], [43], [44], [45]]. Another possible explanation is that we not only included patients with a newly diagnosed thyrotoxicosis but also patients with subclinical hyperthyroidism that may still be very early in their course of disease.

In patients with other (thyroid) disease discordant results were mostly seen in patients who only showed antibody positivity in the TBII-assay. This can be explained by the high proportion of patients with TBII-positive outcomes in the grey zone for positivity [46]. Indeed, most of these discordant results disappeared when we applied a higher cut-off value for negativity (2-or 3-times the normal cut-off of 2.9). The high proportion of patients with (false) positive results in this grey zone is also underlined by a study from Smit et al., where they suggest a more accurate cut-off of 4.5 IU/L in patients with newly diagnosed GD using the ELiA TBII-assay [47]. Furthermore, a recent letter from the manufacturer, which was send to our center after we completed our study, announced technical improvement of the test due to the many false positives in the low positive range (2.9–5 IU/L). By using two or three times the cut-off, which is based on the Dutch guideline for obstetrics and gynecology and the international guidelines for pregnant women where they recommend close monitoring when the TRAb titer is more than 2–3 times the cut-off, we aimed to exclude most false positive results and improve clinical relevance [23,24]. More detailed analysis of the remaining outliers in the subgroup of patients with other (thyroid) disease, revealed TRAb positivity mainly in serum of patients with a thyroiditis. This is in line with previous literature where TRAb positivity is described in patients with (autoimmune) thyroiditis [[48], [49], [50]].

In case of active GO most patients showed TRAb positivity in both assays. However, 2 active GO patients (10.5 %) were TSI positive but TBII negative. This latter observation corroborates previous studies that demonstrated a more pronounced correlation between TSI and clinical signs of GO activity [17,18,51,52]. Furthermore, there were 4 patients with a negative TRAb result in both assays. This could be explained by the fact that some patients are referred to our academic center in a later stage of the treatment course or where antibody negative from the start.

The strength of our study is the relatively large sample size (N = 356). The inclusion of patients at different stages in their disease and treatment course can been seen as a limitation of the study. In our academic thyroid center we see mostly patients that are referred to us by other hospitals due to complicated disease course and are thus more often in a later disease stage. This is also the reason we only had 31 newly diagnosed treatment naïve patients included in our study. As we included all patients who had TBII measurement in the selected time period and not only patients presenting with a (new) hyperthyroidism, applying the results to a general population is difficult. This, and also the retrospective nature of the study, could influence the outcome of the results by the presence of selection bias. However, as our Laboratory staff was not aware of patient history or diagnosis, the inclusion and analysis of both assays was completely blinded.

Binding of TSH-R stimulating antibodies is restricted to the N-terminus of the TSH-R extracellular domain, while there is more epitope heterogeneity found across the TSH-R extracellular domain for the blocking antibodies [[55], [56], [57], [58], [59], [60], [61]]. In line with this, is the observation that although the automated bridge-based binding assay was originally developed for the specific detection of TSI, there are several studies that show positivity of some sera containing functional TSH-R blocking antibodies (but not all sera with functional blocking antibodies) in this bridge-based TSI binding assay [27,29,30]. Considering the fact that patients can produce a mixture of TSH-R stimulating and blocking antibodies at the same time or that a shift between stimulating and blocking antibodies (or vice versa) may occur, knowledge on biological activity and not simply presence/absence of TSH-R Abs is important and as such functional bioassays are more accurate [25,26,29,30,62]. However, these bioassays are technically more complex and not routinely implemented for clinical use in our and most other hospitals. In conclusion, in our academic thyroid center the diagnostic performance of the TSI-assay seems better than the TBII-assay with a higher overall specificity, PPV, NPV and DOR. Using a higher cut-off value can be helpful in assessing clinical relevance.

## Funding statement

This research did not receive any funding.

## Ethics statement

This retrospective clinical practice study was reviewed and approved by the medical ethical committee of Erasmus MC (MEC-2016-606). Participants provided consent to participate in the study. All authors declare that the study complies with all regulations.

## Data availability statement

The data associated with our study has not been deposited into a publicly available repository. Data are available on request.

## CRediT authorship contribution statement

**Mathé van Balkum:** Conceptualization, Data curation, Formal analysis, Investigation, Methodology, Software, Visualization, Writing – original draft, Writing – review & editing. **Marco W.J. Schreurs:** Conceptualization, Data curation, Investigation, Methodology, Project administration, Resources, Supervision, Validation, Visualization. **W. Edward Visser:** Conceptualization, Data curation, Supervision, Writing – review & editing. **Robin P. Peeters:** Conceptualization, Data curation, Methodology, Resources, Supervision, Validation, Writing – review & editing. **Willem A. Dik:** Conceptualization, Data curation, Investigation, Methodology, Project administration, Software, Supervision, Validation, Visualization, Writing – original draft, Writing – review & editing.

## Declaration of competing interest

The authors declare that they have no known competing financial interests or personal relationships that could have appeared to influence the work reported in this paper.

## References

[1] Kahaly G.J., Bartalena L., Hegedus L., Leenhardt L., Poppe K., Pearce S.H. (2018). 2018 European thyroid association guideline for the management of graves' hyperthyroidism. Eur. Thyroid J..

[2] Ross D.S., Burch H.B., Cooper D.S., Greenlee M.C., Laurberg P., Maia A.L., Rivkees S.A., Samuels M., Sosa J.A., Stan M.N., Walter M.A. (2016). 2016 American thyroid association guidelines for diagnosis and management of hyperthyroidism and other causes of thyrotoxicosis. Thyroid.

[3] Brent G.A. (2008). Clinical practice. Graves' disease. N. Engl. J. Med..

[4] Hussain Y.S., Hookham J.C., Allahabadia A., Balasubramanian S.P. (2017). Epidemiology, management and outcomes of Graves' disease-real life data. Endocrine.

[5] Cooper D.S. (2003 Aug 9). Hyperthyroidism. Lancet.

[6] Davies T.F., Andersen S., Latif R., Nagayama Y., Barbesino G., Brito M., Eckstein A.K., Stagnaro-Green A., Kahaly G.J. (2020 Jul 2). Graves' disease. Nat Rev Dis Primers.

[7] Evans M., Sanders J., Tagami T., Sanders P., Young S., Roberts E., Wilmot J., Hu X., Kabelis K., Clark J., Holl S., Richards T., Collyer A., Furmaniak J., Smith B.R. (2010 Sep). Monoclonal autoantibodies to the TSH receptor, one with stimulating activity and one with blocking activity, obtained from the same blood sample. Clin. Endocrinol..

[8] Miyauchi A., Amino N., Tamaki H., Kuma K. (1988 Sep). Coexistence of thyroid-stimulating and thyroid-blocking antibodies in a patient with Graves' disease who had transient hypothyroidism. Am. J. Med..

[9] Fort P., Lifshitz F., Pugliese M., Klein I. (1988 Mar). Neonatal thyroid disease: differential expression in three successive offspring. J. Clin. Endocrinol. Metab..

[10] Cho B.Y., Shong M.H., Yi K.H., Lee H.K., Koh C.S., Min H.K. (1992 Jun). Evaluation of serum basal thyrotrophin levels and thyrotrophin receptor antibody activities as prognostic markers for discontinuation of antithyroid drug treatment in patients with Graves' disease. Clin. Endocrinol..

[11] Struja T., Kaeslin M., Boesiger F., Jutz R., Imahorn N., Kutz A., Bernasconi L., Mundwiler E., Mueller B., Christ-Crain M., Meienberg F., Ebrahimi F., Henzen C., Fischli S., Kraenzlin M., Meier C., Schuetz P. (2017 Apr). External validation of the GREAT score to predict relapse risk in Graves' disease: results from a multicenter, retrospective study with 741 patients. Eur. J. Endocrinol..

[12] Vos X.G., Endert E., Zwinderman A.H., Tijssen J.G.P., Wiersinga WMl (2016 Apr). Predicting the risk of Recurrence before the start of antithyroid drug therapy in patients with graves' hyperthyroidism. J. Clin. Endocrinol. Metab..

[13] Schott M., Morgenthaler N.G., Fritzen R., Feldkamp J., Willenberg H.S., Scherbaum W.A., Seissler J. (2004 Feb). Levels of autoantibodies against human TSH receptor predict relapse of hyperthyroidism in Graves' disease. Horm. Metab. Res..

[14] Khoo D.H., Ho S.C., Seah L.L., Fong K.S., Tai E.S., Chee S.P., Eng P.H., Aw S.E., Fok A.C. (1999 Dec). The combination of absent thyroid peroxidase antibodies and high thyroid-stimulating immunoglobulin levels in Graves' disease identifies a group at markedly increased risk of ophthalmopathy. Thyroid.

[15] Kahaly G.J., Wuster C., Olivo P.D., Diana Tl (2019 Jul 1). High titers of thyrotropin receptor antibodies are associated with orbitopathy in patients with graves disease. J. Clin. Endocrinol. Metab..

[16] Dik W.A., Virakul S., van Steensel L. (2016 Jan). Current perspectives on the role of orbital fibroblasts in the pathogenesis of Graves' ophthalmopathy. Exp. Eye Res..

[17] George A., Diana T., Langericht J., Kahaly G.J. (2021 Feb 2). Stimulatory thyrotropin receptor antibodies are a Biomarker for graves' orbitopathy. Front. Endocrinol..

[18] Bluszcz G.A., Bednarczuk T., Bartoszewicz Z., Kondracka A., Walczak K., Zurecka Z., Demkow U., Miskiewics P. (2018). Clinical utility of TSH receptor antibody levels in Graves' orbitopathy: a comparison of two TSH receptor antibody immunoassays. Cent. Eur. J. Immunol..

[19] Gerding M.N., van der Meer J.W., Broenink M., Bakker O., Wiersinga W.M., Prummel M.F. (2000 Mar). Association of thyrotrophin receptor antibodies with the clinical features of Graves' ophthalmopathy. Clin. Endocrinol..

[20] Tvito-Green R., Reich E., Robenshtok E., Chodick G., Ron-Kella Y., Stiebel-Kalish H. (2014 Feb). Third-generation ELISA thyrotropin-receptor antibody levels as an adjuvant tool to guide management of patients with Graves orbitopathy. Endocr. Pract..

[21] Kampmann E., Diana T., Kanitz M., Hoppe D., Kahaly G.J. (2015). Thyroid stimulating but not blocking autoantibodies are highly prevalent in severe and active thyroid-associated orbitopathy: a prospective study. Internet J. Endocrinol..

[22] Abeillon-du Payrat J., Chikh K., Bossard N., Bretones P., Gaucherand P., Claris O., Charrié A., Raverot V., Orgiazzi J., Borson-Chazot F., Bournaud C. (2014 Oct). Predictive value of maternal second-generation thyroid-binding inhibitory immunoglobulin assay for neonatal autoimmune hyperthyroidism. Eur. J. Endocrinol..

[23] Laurberg P., Nygaard B., Glinoer D., Grussendorf M., Orgiazzi J. (1998 Dec). Guidelines for TSH-receptor antibody measurements in pregnancy: results of an evidence-based symposium organized by the European Thyroid Association. Eur. J. Endocrinol..

[24] Stagnaro-Green A., Abalovich M., Alexander E., Azizi F., Mestman J., Negro R., Nixon A., Pearce E.N., Soldin O.P., Sullivan S., Wiersinga W. (2011 Oct). American thyroid association taskforce on thyroid disease during pregnancy and postpartum. Guidelines of the American thyroid association for the diagnosis and management of thyroid disease during pregnancy and postpartum. Thyroid.

[25] Kahaly G.J., Diana T., Olivo P.D. (2020 Jan). TSH receptor antibodies: relevance & utility. Endocr. Pract..

[26] Diana T, Kanitz M, Lehmann M, Li Y, Olivo PD & Kahaly GJ. Standardization of a bioassay for thyrotropin receptor stimulating autoantibodies. Thyroid 2015x25(2):169-175. DOI: 10.1089/thy.2014.0346. 10.1089/thy.2014.0346 25317659

[27] Frank C.U., Braeth S., Dietrich J.W., Wanjura D., Loos U. (2015 Nov). Bridge technology with TSH receptor chimera for sensitive direct detection of TSH receptor antibodies causing graves' disease: analytical and clinical evaluation. Horm. Metab. Res..

[28] Tozzoli R., D'Aurizio F., Villalta D., Giovanella L. (2017 Jan 1). Evaluation of the first fully automated immunoassay method for the measurement of stimulating TSH receptor autoantibodies in Graves' disease. Clin. Chem. Lab. Med..

[29] Allelein S., Diana T., Ehlers M., Kanitz M., Hermsen D., Schott M., Kahaly G.J. (2019 Jun). Comparison of a bridge immuno- assay with two bioassays for thyrotropin receptorantibody detection and differentiation. Horm. Metab. Res..

[30] Diana T., Wüster C., Olivo P.D., Unterrainer A., König J., Kanitz M., Bossowski A., Decallonne B., Kahaly G.J. (2017 Sep). Performance and specificity of 6 immunoassays for tsh receptor antibodies: a multicenter study. Eur. Thyroid J..

[31] Allelein S., Ehlers M., Goretzki S., Hermsen D., Feldkamp J., Haase M., Dringenberg T., Schmid C., Hautzel H., Schott M. (2016 Dec). Clinical evaluation of the first automated assay for the detection of stimulating TSH receptor autoantibodies. Horm. Metab. Res..

[32] Autilio C., Morelli R., Locantore P., Pontecorvi A., Zuppi C., Carrozza C. (2018 Jan). Stimulating TSH receptor autoantibodies immunoassay: analytical evaluation and clinical performance in Graves' disease. Ann. Clin. Biochem..

[33] Diana T., Wuster C., Kanitz M., Kahaly G.J. (2016 Oct). Highly variable sensitivity of five binding and two bio-assays for TSH-receptor antibodies. J. Endocrinol. Invest..

[34] Kim J.J., Jeong S.H., Kim B., Kim D., Jeong S.H. (2019 Oct). Analytical and clinical performance of newly developed immunoassay for detecting thyroid-stimulating immunoglobulin, the Immulite TSI assay. Scand. J. Clin. Lab. Invest..

[35] Scappaticcio L., Trimboli P., Keller F., Imperiali M., Piccardo A., Giovanella L. (2020 Feb). Diagnostic testing for Graves' or non-Graves' hyperthyroidism: a comparison of two thyrotropin receptor antibody immunoassays with thyroid scintigraphy and ultrasonography. Clin. Endocrinol..

[36] Silvestre R.A., Lafuente A.A., Jiménez-Mendiguchía L., Garcia-Cano A., Lopez R.R., Garcia-Izquierdo B., Pardo de Santayana C., Iglesias P., Diez J., Gomez A., Bernabeu-Andreu F.A. (2021 Mar 2). Comparison of three methods for determining anti-thyrotropin receptor antibodies (TRAb) for diagnosis of Graves' disease: a clinical validation. Adv Lab Med.

[37] Struja T., Jutzi R., Imahorn N., Kaeslin M., Boesiger F., Kutz A., Mundwiler E., Huber A., Kraenzlin M., Mueller B., Meier C., Bernasconi L., Schuetz P. (2019 Apr 25). Comparison of Five TSH-Receptor Antibody Assays in Graves' disease: results from an observational pilot study. BMC Endocr. Disord..

[38] Bartalena L., Baldeschi L., Dickinson A., Eckstein A., Kendall-Taylor P., Marcocci C., Mourits M., Perror P., Boboridis K., Boschi A., Curro N., Daumerie C., Kahaly G.J., Krassas G.E., Lane C.M., Lazarus J.H., Marino M., Nardi M., Neoh C., Orgiazzi J., Pearce S., Pinchera A., Pitz S., Salvi M., Sivelli P., Stahl M., von Arx G., Wiersinga W.M. (2008). European group on graves' orbitopathy (EUGOGO). Consensus statement of the European group on graves' orbitopathy (EUGOGO) on management of GO. Eur. J. Endocrinol..

[39] Glas A.S., Lijmer J.G., Prins M.H., Bonsel G.J., Bossuyt P.M.M. (2003 Nov). The diagnostic odds ratio: a single indicator of test performance. J. Clin. Epidemiol..

[40] NVOG (2010). Richtlijn schildklier en zwangerschap. https://www.nvog.nl/wp-content/%20uploads/2017/12/Schildklier-en-zwangerschap-2.0-04-06-2010.pdf.

[41] De Groot L., Abalovich M., Alexander E.K., Amino N., Barbour L., Cobin R.H., Eastman C.J., Lazarus J.H., Luton D., Mandel S.J., Mestman J., Rovet J., Sullivan S. (2012 Aug). Management of thyroid dysfunction during pregnancy and postpartum: an Endocrine Society clinical practice guideline. J. Clin. Endocrinol. Metab..

[42] Ilicki A., Gamstedt A., Karlsson F.A. (1992 May). Hyperthyroid Graves' disease without detectable thyrotropin receptor antibodies. J. Clin. Endocrinol. Metab..

[43] Kawai K., Tamai H., Matsubayashi S., Mukuta T., Morita T., Kubo C., kuma K. (1995 Nov). A study of untreated Graves' patients with undetectable TSH binding inhibitor immunoglobulins and the effect of anti-thyroid drugs. Clin. Endocrinol..

[44] Suziki N., Noh J.Y., Kameda T., Yoshihara A., Ohye H., Suzuki M., Matsumoto M., Kunii Y., Iwaku K., Watanebe N., Mukasa K., Kozaki A., Inoue T., Sugino K., Ito K. (2018 Apr 19). Clinical course of thyroid function and thyroid associated-ophthalmopathy in patients with euthyroid Graves' disease. Clin. Ophthalmol..

[45] Paunkovic J., Pankovic J. (2006 Jan). Does autoantibody-negative Graves' disease exist? A second evaluation of the clinical diagnosis. Horm. Metab. Res..

[46] Luther T., Sjolander S., Kast M., Konrad C., Inc T.F.S. (2016). White Paper: EliA Anti-TSH-R Assay.

[47] Smit M.A., van Kinschot C.M.J., van der Linden J., van Noord C., Kos S. (2020 Mar). Measurement of anti-TSH receptor antibodies: what is the correct cut-off value. Neth. J. Med..

[48] Morita T., Tamai H., Oshima A., Mukuta T., Fukata S., Kuma K., Kumagai L.F., Nagataki S. (1990 Oct). The occurrence of thyrotropin binding-inhibiting immunoglobulins and thyroid-stimulating antibodies in patients with silent thyroiditis. J. Clin. Endocrinol. Metab..

[49] Iitaka M., Nomotani N., Hisaoka T., Noh J.Y., Ishikawa N., Ishii J., Katayama S., Ito K. (1998 Apr). TSH receptor antibody-assiocated thyroid dysfunction following subacute thyoidititis. Clin. Endocrinol..

[50] Kahaly G.J., Diana T., Glang J., Kanitz M., Pitz S., Konig J. (2016 May). Thyroid stimulating antibodies are highly prevalent in Hashimoto's thyroiditis and associated Orbitopathy. J. Clin. Endocrinol. Metab..

[51] Noh J.Y., Hamada N., Inoue Y., Abe Y., Ito K., Ito K. (2000 Sep). Thyroid-stimulating anyibody is related to Graves' ophtalmopathy, but thyrotropin-binding inhibitor immunoglobulin is related to hyperthyroidism in patients with Graves' disease. Thyroid.

[52] Jang S.Y., Shin D.Y., Lee E.J., Choi Y.J., Lee S.Y., Yoon J.S. (2013 Jul). Correlation between TSH receptor antibody assays and clinical manifestations of Graves' orbitopathy. Yonsei Med. J..

[53] Villagelin D., Santos R.B., Romaldini J.H. (2020). Remission rate of graves’ disease and the trend of changes in serum TSH receptor antibodies in prolonged antithyroid drug treatment. J. Clin. Endocrinol. Metab..

[54] Laurberg P., Wallin G., Tallstedt, Abraham-Nordlling M., Lundell G., Tørring O. (2008 Jan). TSH-receptor autoimmunity in Graves’disease after therapy with anti-thyroid drugs, surgery or radioiodine: a 5-year prospective randomized study. Eur. J. Endocrinol..

[55] Sanders J., Chirgadze D.Y., Sanders P., Baker S., Sullivan A., Bhardwaja A., Bolton J., Reeve M., Nakatake N., Evans M., Richards T., Powell M., Núñez Miguel R., Blundell T.L., Furmaniak J., Rees-Smith B. (2007 May). Thyroid.

[56] Furmaniak J., Sanders J., Rees-Smith B. (2012 Mar 21). Blocking type TSH receptor antibodies. Auto Immun Highlights.

[57] Rees Smith B., Sanders J., Evans M., Tagami T., Furmaniak J. (2009 Jun). TSH receptor-autoantibody interactions. Horm. Metab. Res..

[58] Ando T., Latif R., Daniel S., Eguchi K., Davies T.F. (2004 Nov). Dissecting linear and conformational epitopes on the native thyrotropin receptor. Endocrinology.

[59] Minich W.B., Lenzner C., Bergmann A., Morgenthaler N.G. (2004 Jan). A coated tube assay for the detection of blocking thyrotropin receptor autoantibodies. J. Clin. Endocrinol. Metab..

[60] Grasso Y.Z., Kim M.R., Faiman C., Kohn L.D., Tahara K., Gupta M.K. (1999 Jun). Epitope heterogeneity of thyrotropin receptor-blocking antibodies in Graves' patients as detected with a wild-type versus chimeric thyrotropin receptors. Thyroid.

[61] Morgenthaler N.G., Chin Ho S., Minich W.B. (2007 Mar). Stimulating and blocking thyroid-stimulating hormone (TSH) receptor autoantibodies from patients with Graves' disease and autoimmune hypothyroidism have very similar concentration, TSH receptor affinity, and binding sites. J. Clin. Endocrinol. Metab..

[62] Evans M., Sanders J., Tagamit T., Sanders P., Young S., Roberts E., Wilmot J., Hu X., Kabelis K., Clark J., Holl S., Richards T., Collyer A., Furmaniak J., Rees-Smith B. (2010 Sep). Monoclonal autoantibodies to the TSH receptor, one with stimulating activity and one with blocking activity, obtained from the same blood sample. Clin. Endocrinol..

