# Supplementary material for: Comparison of two different TSH-receptor antibody assays: A clinical practice study
Source: Heliyon. 2023 Nov 18;9(12):e22468. doi: 10.1016/j.heliyon.2023.e22468 (PMC10724564; doi:10.1016/j.heliyon.2023.e22468)
Supplement: Multimedia component 1 [file mmc1.docx]

**Supplemental tables**

**Supplemental table 1.** Characteristics of subgroup with other (thyroid) disease

| **Other diagnosis** | **N (%)** |
| --- | --- |
| Thyroiditis | 31 (18.0%) |
| Pregnancy related | 4 (2.3%) |
| Fertility work-up gynecologist | 19 (11.0%) |
| Multinodular goiter | 21 (12.2%) |
| AIT | 13 (7.6%) |
| Hypothyroidism/M. Hashimoto | 6 (3.5%) |
| Work-up subclinical hyper/hypothyroidism | 10 (5.8%) |
| Oversuppletion LT4 | 2 (1.2%) |
| Iodinated contrast | 4 (2.3%) |
| Otherwise | 62 (36.0%) |

**Supplemental table 2a.** Serological outcomes in total study population using two times the cut-off for negativity^a^

|  | **M. Graves (n=90)** | **M. Graves with GO (N=94)** | **Other diagnosis (N=172)** |
| --- | --- | --- | --- |
| TBII and TSI both negative | 37 (41.1%) | 43 (45.7%) | 166 (96.5%) |
| TBII and TSI both positive | 32 (35.6%) | 32 (34.0%) | 3 (1.7%) |
| TBII is positive and TSI is negative | 1 (1.1%) | 2 (2.1%) | - |
| TBII is negative and TSI is positive | 20 (22.2%) | 17 (18.1%) | 3 (1.7%) |
| **Outcome** | Concordant 69/90 = 76.7%  Discrepancy 21/90 = 23.3% | Concordant 75/94 = 79.8%  Discrepancy 19/94 = 20.2% | Concordant 169/172 = 98.3%  Discrepancy 3/ 172 = 1.7% |

^a^ Two times cut-off for TBII assay (ELiA) <5.8 IU/L and TSI-assay (Immulite) <1.1 IU/L.

**Supplemental table 2b.** Serological outcomes in total study population using three times the cut-off for negativity^a^

|  | **M. Graves (n=90)** | **M. Graves with GO (N=94)** | **Other diagnosis (N=172)** |
| --- | --- | --- | --- |
| TBII and TSI both negative | 45 (50%) | 51 (54.3%) | 167 (97.1%) |
| TBII and TSI both positive | 23 (25.6%) | 27 (28.7%) | 2 (1.2%) |
| TBII is positive and TSI is negative | - | 1 (1.1%) | - |
| TBII is negative and TSI is positive | 22 (24.2%) | 15 (16.0%) | 3 (1.7%) |
| **Outcome** | Concordant 68/90 = 75.6%  Discrepancy 22/90 = 24.4% | Concordant 78/94 = 83.0%  Discrepancy 16/94 = 17.0% | Concordant 169/172 = 98.3%  Discrepancy 3/172 = 1.7% |

^a^ Three times cut-off for TBII assay (ELiA) <8.7 IU/L and TSI-assay (Immulite) <1.65 IU/L.

**Supplemental table 3. Outliers other (thyroid) disease^a^**

|  | **TBII IU/L** | **TSI IU/L** | **TSH mU/L** | **Free T4 pmol/L** |
| --- | --- | --- | --- | --- |
| 1. Toxic MNG and RAI | 15.0 | 5.74 | 0.003 | 27.3 |
| 2. Thyroiditis and Alemtuzumab | 358 | >40 | 0.453 | 31.3 |
| 3. Thyroiditis | 8.7 | 3.98 | 127.9 | 5.4 |
| 4. Thyroiditis | 1.5 | 2.96 | 0.02 | 19.3 |
| 5. Fertility work-up | 2.7 | 1.15 | 2.66 | 28.8 |
| 6. Thyroiditis and immunotherapy | 4.6 | 2.57 | 44.7 | 12.9 |

**^a^** Outliers in the other (thyroid) disease group
